# Causes, consequences, and policy responses to the migration of health workers: key findings from India

**DOI:** 10.1186/s12960-017-0199-y

**Published:** 2017-04-05

**Authors:** Margaret Walton-Roberts, Vivien Runnels, S. Irudaya Rajan, Atul Sood, Sreelekha Nair, Philomina Thomas, Corinne Packer, Adrian MacKenzie, Gail Tomblin Murphy, Ronald Labonté, Ivy Lynn Bourgeault

**Affiliations:** 1 Balsillie School of International Affairs, 67 Erb Street West, Waterloo, ON N2L 6C2 Canada; 2 Faculty of Medicine, University of Ottawa, 850 Peter Morand Crescent, Ottawa, ON K1G 3Z7 Canada; 3 Centre for Development Studies, Prasanth Nagar, Medical College P.O, Ulloor, Thiruvananthapuram, 695 011 Kerala India; 4 Centre for the Study of Regional Development, School of Social Sciences, JNU, Delhi, India; 5 Athiyara Madom Devi Temple Lane, Vanchiyoor, Thiruvananthapuram, 695035 Kerala India; 6 College of Nursing, All India Institute of Medical Sciences, New Delhi, India; 7 WHO/PAHO Collaborating Centre on Health Workforce Planning and Research, Dalhousie University, 5869 University Avenue, Halifax, Nova Scotia B3H 4R2 Canada; 8 WHO/PAHO Collaborating Centre on Health Workforce Planning and Research, School of Nursing, Faculty of Health Professions, Dalhousie University, 5869 University Avenue, Halifax, Nova Scotia B3H 4R2 Canada; 9 School of Epidemiology, Public Health and Preventive Medicine Faculty of Medicine, University of Ottawa, 850 Peter Morand Crescent, Ottawa, ON K1G 3Z7 Canada; 10 Telfer School of Management, University of Ottawa, 1 Stewart Street, Ottawa, ON K1N 6N5 Canada

**Keywords:** Health worker migration, Human resources for health, India, Doctors, Nurses, Causes, Consequences, Policy responses

## Abstract

**Background:**

This study sought to better understand the drivers of skilled health professional migration, its consequences, and the various strategies countries have employed to mitigate its negative impacts. The study was conducted in four countries—Jamaica, India, the Philippines, and South Africa—that have historically been “sources” of health workers migrating to other countries. The aim of this paper is to present the findings from the Indian portion of the study.

**Methods:**

Data were collected using surveys of Indian generalist and specialist physicians, nurses, midwives, dentists, pharmacists, dieticians, and other allied health therapists. We also conducted structured interviews with key stakeholders representing government ministries, professional associations, regional health authorities, health care facilities, and educational institutions. Quantitative data were analyzed using descriptive statistics and regression models. Qualitative data were analyzed thematically.

**Results:**

Shortages of health workers are evident in certain parts of India and in certain specialty areas, but the degree and nature of such shortages are difficult to determine due to the lack of evidence and health information. The relationship of such shortages to international migration is not clear. Policy responses to health worker migration are also similarly embedded in wider processes aimed at health workforce management, but overall, there is no clear policy agenda to manage health worker migration. Decision-makers in India present conflicting options about the need or desirability of curtailing migration.

**Conclusions:**

Consequences of health work migration on the Indian health care system are not easily discernable from other compounding factors. Research suggests that shortages of skilled health workers in India must be examined in relation to domestic policies on training, recruitment, and retention rather than viewed as a direct consequence of the international migration of health workers.

## Background

The global migration of health workers remains an issue of concern for health provision and development [1, 2]. India is a major source country (exporter) of health workers, but determining how skilled health worker migration interacts with the Indian health care system is challenging to measure, especially in terms of relationships of causation. Although there are some studies of Indian health worker migration [3–6], comprehensive data and analysis on this issue are generally lacking in much of the current research. We do know that Indian-trained health workers have played an important role in serving the needs of overseas health systems since the 1950s [7]. India remains one of the largest exporters of all health professionals, it is the world’s largest supplier of physicians [4], and the emigration of nurses is also significant, with estimates ranging from 20 to 50% of Indian nursing program graduates intending to seek overseas opportunities [5, 6]. Health worker migration (HWM) has thus been assumed to be associated with shortages and uneven distribution of health workers. The costs and human capital losses associated with health worker migration are presumed to be significant for India, but, as we outline below, they have not been fully assessed in terms of overall numerical or policy significance.

Our four-country study of “source” country for health professional migration included South Africa [8], Jamaica [9], India, and the Philippines, countries that have historically supplied trained health workers for other countries. We set out to investigate the causes, consequences, and policy responses emerging from the international migration of health workers. In this paper, we investigate these issues in the Indian context, with specific reference to the states of Kerala and Punjab.

### Indian health and health care context

India has a population of 1.26 billion, about 17.5% of the world’s population [10, 11]. It carries 25% of the global burden of child deaths and 20% of global maternal deaths [12] . India is undergoing both a demographic transition as a result of increased life expectancy and decreased death and birth rates and an epidemiological transition in which non-communicable diseases, such as heart disease and diabetes are rapidly replacing infectious diseases as the major cause of mortality [13]. Globalization and rapid economic change have also led to significant social change including the expansion of the middle class alongside increasing inequality, which together have both enhanced and changed the demands placed on India’s health system [14]. International migration and accompanying financial remittances have increased in parallel with globalization tendencies, which is a significant policy issue for India considering it was the largest recipient of migrant remittances in 2013 (70 billion US dollars), and by 2050 is projected to emerge as one of the world’s largest migrant-sending countries [15].

Access to health care is heavily influenced by social class and geographical location, with rural areas being generally underserved [16]. India’s public health system is meant to serve the poor majority of India’s population; however, the private health sector is now playing an increased role in health service delivery as well as health care training and education, such that the Indian health care system has come to be defined by the extensive involvement of the private sector [17]. The private sector owns 60% of hospitals and 75% of dispensaries and employs 80% of all qualified doctors in India [18].

The public health sector in India is ranked 6th lowest worldwide in terms of percent of GDP invested in health (0.9%), but it is among the top 20 countries in terms of private expenditure on health (including out-of-pocket expenditures), accounting for 4.2% of GDP [17]. India’s 2015 Draft National Health Policy does not show any deviation from this path of privatization, rather there are signs that the private sector will continue to penetrate public sector health infrastructure including the aforementioned increased role in health care training and education [19].

In light of both domestic and international demands for health professionals, the health worker training sector in India has experienced significant growth, mainly through private corporate investment [20]. Growth in this sector has also been driven by an interest in overseas-oriented employment opportunities [21, 22]. This rapid growth has raised concerns about the quality of training and the level of corruption in the regulation of the sector [23]. In this regard, the Indian health educational sector exhibits the same tendencies underway in the health care sector, which has seen annual growth rates of approximately 14% per annum this decade, and is estimated to hit 21% next decade, driven mostly by corporate investment. The Indian government has played a facilitative role in corporate health care; the “Government has had an active policy in the last 25 years of building a positive economic climate for the health care industry” [24], p. 9. The corporate and global orientation of health care service and training might incentivize health worker migration at the cost of slowing National Health Policy reforms. In that regard, understanding how the two interact remains a key research issue.

As with many developing economies, India faces several health workforce challenges, including a lack of high-quality training, geographic maldistribution of workers, and loss of workers to overseas destinations [25]. Indeed, India has been considered to be a “crisis” country with respect to all health workforce stocks as identified by the 2006 World Health Report [21, 26, 27] and, with Jamaica, was one of the two countries in our four-country study in which the density of physicians, midwives, and nurses fell below the WHO recommended minimum of 22.8 total midwives, nurses, and physicians per 10 000 population [28, 29] (see Table 1).

**Table 1:** Health worker density for study countries of India, Jamaica, the Philippines, South Africa and for comparison purposes, the USA (available data). (Source: Global Health Observatory data repository density per 1000) accessed, December 7th 2015 http://apps.who.int/gho/data/node.main.A1444?lang=en

Country	Year	Physicians density (per 1 000 population)	Nursing and midwifery personnel density (per 1 000 population)	Dentistry personnel density (per 1 000 population)	Pharmaceutical personnel density (per 1 000 population)	Laboratory health workers density (per 1 000 population)	Other health workers density (per 1 000 population)
India	2011	0.743	1.711	0.095	0.529		0.506
Jamaica	2008	0.411	1.09	0.09	0.06	0.21	0.42
Philippines	2011				0.886		
South Africa	2011	0.758	4.72	0.192	0.369	0.153	2.111
United States of America	2011	2.452					8.459

Health worker data produced by India must be interpreted with caution. The diversity of India’s health workers (some of whose practice roles may have been specifically developed to respond to local need), India’s different systems of medicine (allopathic, Ayurveda, Yoga, Unani, Sidha, and homeopathy), its public and private sector health care and significant regional differences make it difficult to collect data consistently and present a robust data picture with regard to health workforce and health worker migration [30, 31]. Data on annual production from educational institutions is available, but live professional registration data varies by state (some states do not have professional regulatory bodies) and there may be double counting when internal state-to-state migration occurs [32]. Furthermore, regulatory bodies’ data collection and conservation records are weak in India: when health workers migrate, there are few if any efforts by the different regulatory bodies to conserve or maintain continuous records. The reliability of Indian health worker data is also questionable [31], with many non-licensed, non-regulated, and non-qualified persons practicing medicine in India. Therefore, the baseline assumptions with regard to India’s health workforce in terms of qualified workers and skills, and the full extent of health worker migration, are unclear.

## Methods

This study sought to better understand the drivers of skilled health professional migration in four source (sending) countries, its consequences, and the various strategies countries have employed to mitigate its negative impacts. The project used the same mixed method approach for each country, comprising a scoping review of published literature on health worker migration, a survey of health workers, and interviews with key stakeholders. In this paper, we report on the India case. All study activities were facilitated collaboratively by the study’s Principal Investigators at the University of Ottawa and Dalhousie University in Canada and co-investigators in Canada (Wilfrid Laurier University) and India (the Centre for Development Studies in Trivandrum, Kerala, and the Institute for Development and Communication in Chandigarh, Punjab). The study was approved by the University of Ottawa (Ethics Approval Certificate numbers H07-10-02H and H07-10-02C). In India, ethics clearance was granted by the Institute of Development Communication (Punjab) and the Centre for Development Studies (Kerala).

### Site selection

In order to operationalize the research and recognize the complexity of data collection at the national scale in India, we focused our empirical research in two states. In terms of regional differences, Kerala has long been seen as the main source location for nurse outmigration [33], and Punjab has seen a substantial increase in training capacity, especially for nurses, for export [34]. Kerala and Punjab were chosen due to the states’ level of health care training capacity and their high degree of outward orientation in terms of international migration; Kerala and Punjab are in the top three Indian states with the highest share of total emigrants (outmigrants) residing in another country [35].

### Scoping review

The scoping review of the literature followed the process developed by Arksey and O’Malley [36], using the medical subject headings (MeSH) terms “migration,” “health professionals”/“health workers”/“nurses”/“physicians,” and “India” in a search of PubMed (including MEDLINE) and Embase databases. We also undertook a search and analysis of the published and gray literature and public domain information from a number of stakeholder websites, including Indian government agencies. Articles were included if they addressed one or more of the three research questions, focused on India, and were published between 2000 and 2012. After inclusion and exclusion criteria were applied (articles were included if they addressed the research questions and mentioned India; articles that related to India but without relevance to the research questions were excluded) and duplicates removed, over 160 documents qualified for analysis. Analysis of the literature was synthesized into a scoping review report, which highlighted our key findings by the following themes: context and overview of the Indian health system; factors influencing migration; implications of migration, and policy responses. The scoping review was employed to set the guidelines and directions for the health worker survey and key stakeholder interview schedules.

### Survey of health workers in Kerala and Punjab

**Fig. 1:**
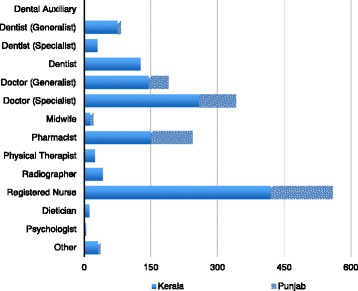
Which one of the following best describes the licensed health profession category to which you belong? (Source Health Professional Migration Survey (*n* = 1719))

The health worker survey targeted doctors, nurses, midwives, dentists, pharmacists, and other health workers. Individuals were invited to participate in the study either through professional association membership or through their place of work. The survey was in English, and the questionnaire was broadly replicated across all four of the project’s study sites. Questions explored respondent’s training, living, and working experiences as well as their views regarding the migration of highly trained health personnel. A total of 1736 Indian health workers completed the survey using a paper-based format in face-to-face encounters, 1337 in Kerala, and 399 in Punjab. Out of this total, 1719 surveys were adequately completed and could be analyzed (see Fig. 1 for the occupational sample comparison). The larger sample in Kerala was a result of our survey being conducted in combination with a broader migration policy institute survey.

In both states in the country, research teams surveyed health workers in urban and peri-urban areas. The survey was completed in south Kerala in Thiruvananthapuram, central Kerala in Ernakulam, and in Kozhikode in the north. Interviews were conducted in both public and private places of work. (A viral infection outbreak in Ernakulam at the time of data collection and health workers’ busy schedules resulted in fewer completed surveys from this region). Respondents in Punjab came from both rural and urban areas across the state and public and private health facilities. Surveys were mostly conducted outside (but near) the worksite at the start or end of shifts. While the survey was written in English, surveyors used English and vernacular languages while administering the survey instrument.

In terms of methods of analysis, quantitative data collected from the surveys were analyzed descriptively. Logistic regression models of respondents’ reports of having taken each of three concrete steps toward migration—applying for foreign residence, applying for a license to practice in a foreign country, and applying for a work permit in a foreign country—were also developed and run separately for each state. These models measured the relationships between having taken these steps and each respondent’s profession, age, sex, and marital status.

### Stakeholder interviews

A total of 74 key stakeholder interviews (KSIs) were carried out in Kerala and Punjab as well as in the Delhi Capital Region where central government health departments are located (28 in Kerala, 27 in Punjab, and 19 in Delhi). In both Kerala and Punjab, KSIs were conducted in English and the local language (Malayalam in Kerala and Punjabi in Punjab). Stakeholders held senior level positions in various health professional associations, state and national governments, education and training organizations and associations, councils dealing with accreditation, registration and regulation of health care professionals, and hospitals (Table 2). The questions focused on the contextual features of Indian health worker migration, what consequences could be attributed to their migration, and what policies were available to address the causes and/or consequences of health worker migration. Dr. Rajan, Dr. Sood, Dr. Nair, Ms. Thomas, and their research assistants conducted all the stakeholder interviews in India.

**Table 2:** Number of key stakeholder interviews by sector (Source Health Professional Migration Interviews) (*n* = 74)

Location	Health care facility	Government	Professional association/council	Recruitment agency	Training/education	Non-governmental organizations
Kerala	4	4	7	3	8	2
Punjab	3	9	6	3	5	1
Delhi	5	2	6	1	5	
Total	12	15	19	7	18	3

Interviews were transcribed in India and analyzed systematically and comprehensively using a common framework developed initially at the University of Ottawa based on the interview schedule questions. Stakeholder responses were coded thematically accordingly to the broad causes, consequences, and responses categories with specific subcodes using Nvivo™ software. Data synthesis was carried out by repeated readings of the data, refinement of the coding scheme, and the development of a comprehensive qualitative and descriptive narrative of the findings for this paper [37]. Because of the critical nature of some of the questions and ensuing discussions, confidentiality of the respondents’ participation in the interviews and anonymity was assured by the researchers. All personal identifiers have been removed in this paper, and the respondents are identified by a letter and number and general category descriptor.

## Results

### Causes of migration

#### Migration intention: who is most likely to migrate?

**Fig. 2:**
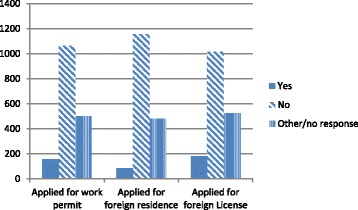
Application for foreign work permit/residence/license (Source Health Professional Migration Survey, *n* = 1719)

**Fig. 3:**
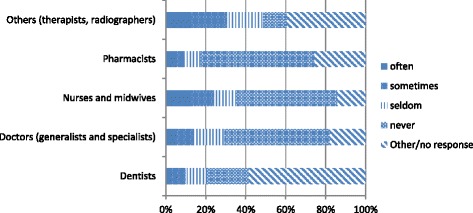
Frequency seeking migration information from recruiters (Source Health Professional Migration Survey, *n* = 1719)

**Fig. 4:**
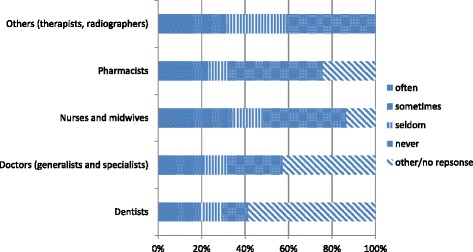
Frequency seeking migration information from personal contacts (Source Health Professional Migration Survey, *n* = 1719)

**Fig. 5:**
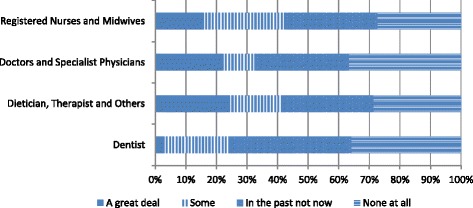
Migration interest among health professionals (Source Health Professional Migration Survey, *n* = 1719)

Evidence from the health worker survey indicates that the majority of health professionals surveyed were not intending to migrate in the next 2 years (of those who responded 75% answered “very unlikely,” 7.5% “very likely”), and the majority had not previously applied for any kind of foreign work permit, residence, or license (see Fig. 2). With regard to information about migration, there was also a fairly strong tendency to never seek out information from recruiters, professional associations, and personal connects, with recruiters the least likely source of information consulted (see Figs. 3 and 4). Approximately 42% of nurses considered migration “a great deal” or “somewhat” compared to 24% of dentists and 32% of doctors. Of all the professions, nurses were the least likely to answer “none at all” about their level of consideration of migration possibilities (27.4% followed by dieticians and other therapists, dentists, and doctors at 36.8%) (see Fig. 5).

Results from the regression models for Kerala showed that, even after controlling for respondents’ age and marital status, their sex and profession were strong predictors of having taken concrete steps to migrate (Tables 3, 4, and 5). Compared to nurses, both general practitioner and specialist physicians were less likely to report having applied for foreign residence, a foreign work permit, or a foreign license to practice. Pharmacists were also less likely than nurses to have applied for a foreign work permit. Male health workers were more likely than females to have applied for foreign residence and to have applied for a foreign work permit. For respondents from Punjab, the strongest predictor of having taken each of these concrete steps toward migration was marital status, with respondents who were single, divorced, separated, or widowed being more likely to have taken steps toward migrating. The relationships with profession and sex found in Kerala were also present but were not statistically significant parameters in the models.

**Table 3:** Odds ratio estimates for respondents reporting having applied for foreign work permit

	Kerala			Punjab		
Effect	Point estimate	95% confidence limits		Point estimate	95% confidence limits	
Dentist vs. nurse	0.421	0.136	1.301	N/A	N/A	N/A
Pharmacist vs. nurse	0.230	0.066	0.808	2.037	0.883	4.696
Generalist physician vs. nurse	0.103	0.023	0.461	1.646	0.602	4.495
Midwife vs. nurse	1.653	0.197	13.853	N/A	N/A	N/A
Specialist physician vs. nurse	0.726	0.312	1.687	1.730	0.744	4.021
Physiotherapist vs. nurse	0.272	0.034	2.184	N/A	N/A	N/A
Radiographer vs. nurse	1.384	0.483	3.965	N/A	N/A	N/A
Male vs. female	2.522	1.389	4.578	1.667	0.797	3.488
Age 25–34 vs. age <24	0.930	0.460	1.882	1.426	0.621	3.275
Age 35–44 vs. age <24	0.512	0.196	1.335	2.568	0.808	8.166
Age 45+ vs. age <24	0.561	0.192	1.644	3.544	1.105	11.365
Married or living together vs. all others	0.799	0.426	1.499	0.508	0.257	1.002

*N/A* insufficient numbers and/or data to include in model

**Table 4:** Odds ratio estimates for respondents reporting having applied for a foreign residence

	Kerala			Punjab		
Effect	Point estimate	95% confidence limits		Point estimate	95% confidence limits	
Dentist vs. nurse	0.528	0.269	1.036	0.468	0.126	1.740
Pharmacist vs. nurse	0.511	0.281	0.929	1.638	0.603	4.451
Generalist physician vs. nurse	0.100	0.041	0.244	1.694	0.732	3.920
Midwife vs. nurse	0.913	0.189	4.395	N/A	N/A	N/A
Specialist physician vs. nurse	0.536	0.300	0.959	1.978	0.860	4.550
Physiotherapist vs. nurse	0.354	0.099	1.262	N/A	N/A	N/A
Radiographer vs. nurse	1.512	0.730	3.132	N/A	N/A	N/A
Male vs. female	1.527	1.022	2.279	1.712	0.821	3.571
Age 25–34 vs. age <24	1.116	0.694	1.795	1.371	0.611	3.076
Age 35–44 vs. age <24	0.629	0.343	1.155	2.425	0.813	7.232
Age 45+ vs. age <24	0.532	0.260	1.090	4.168	1.340	12.965
Married or living together vs. all others	0.430	0.137	1.356	0.520	0.271	0.999

**Table 5:** Odds ratio estimates for respondents reporting having applied for a foreign practice license

	Kerala			Punjab		
Effect	Point estimate	95% confidence limits		Point estimate	95% confidence limits	
Dentist vs. nurse	0.655	0.332	1.293	0.587	0.126	1.740
Pharmacist vs. nurse	0.577	0.311	1.071	1.772	0.876	4.239
Generalist physician vs. nurse	0.182	0.088	0.376	1.515	0.669	4.215
Midwife vs. nurse	0.450	0.055	3.656	N/A	N/A	N/A
Specialist physician vs. nurse	0.496	0.265	0.926	1.812	0.815	4.394
Physiotherapist vs. nurse	1.897	0.701	5.136	N/A	N/A	N/A
Radiographer vs. nurse	1.633	0.776	3.437	N/A	N/A	N/A
Male vs. female	1.245	0.822	1.884	1.700	0.774	3.536
Age 25–34 vs. age <24	0.772	0.461	1.293	1.398	0.642	3.158
Age 35–44 vs. age <24	0.662	0.358	1.224	2.446	0.819	7.937
Age 45+ vs. age <24	0.538	0.263	1.101	3.785	1.291	11.980
Married or living together vs. all others	1.482	0.937	2.343	0.519	0.288	1.082

*N/A* insufficient numbers and/or data to include in model

In terms of the sectors with the greater propensity to provide international migrants, stakeholders indicated agreement that doctors and nurses in the government sector were better paid and had more reason to remain in India. “Doctors or nurses who are serving in government rarely prefer to move out as they feel more secure here than abroad” (A25, Nursing School Representative). Some commented that the nurses in the public sector are actually restricted from going overseas: “Only nurses from the private sector go abroad. [The] government doesn’t allow anyone working in government sector to go. Even those who are working in public sector cannot leave the country without the permission from Ministry of Health nor can they go to other states” (R39, Hospital Medical Head). While the imposition of a moratorium on outmigration from public sector hospitals suggests an important policy, we did not find widespread evidence of this approach being used in practice.

International opportunities for pharmacists were seen as significant, such that even changes in salary and conditions in India would not alter the flow: “Even if you improve working conditions [the] possibility of reduction in migration is very [low]. There are lots of opportunities for pharmacist in foreign countries” (R6, Health Professional Association Representative). The migration process for pharmacists, however, was seen by some to be at the tail end of its recent growth, possibly reflective of a general expansion of pharmacist training in other countries: “Impression is that plenty of pharmacists were migrating, although number is coming down because the foreign markets are flooded with pharmacists” (P17, Pharmacy Association Representative). This suggests an important finding about how fluctuations in international demand drive interest in migration.

Comments with regard to dentists’ emigration suggested increased training of nationals in certain destination countries were influencing the outmigration of Indian-trained health workers: “Earlier we had very good opportunities, because the nationals of those countries were not educated [in large numbers]. But now their nationals are getting educated and as Indians don’t go for a higher degree like PhD, the opportunities are coming down” (R14, Academic Director).

#### Causes of health worker migration

Reasons for Indian health worker migration echo the majority of research findings identified in the scoping review that living and working conditions often combine as key “push” factors [38]. In Kerala, the three work-related factors identified by the majority of respondents as most important in their decisions to migrate were all related to income. These included their income compared to that earned by others in their own country (chosen by 31% of respondents), their income compared to what is needed to enjoy a good quality of life in their country (16%), and their income compared to what they would like to earn (15%). The three living conditions identified by the majority of respondents as most important in their decision to migrate were the cost of living (29%), the ability to find the job they wanted (22%), and the safety of their family (9%). The working conditions that were identified as most important to the decision to migrate that were selected by the fewest respondents (less than 1% of the sample) were as follows: how certain ethnic groups are treated in the workplace (selected by 5 respondents); the risk of contracting a serious illness in the workplace (10 respondents); and respect from management to whom you report (10 respondents). The living conditions identified by the fewest respondents as among their three most important in deciding to migrate were as follows: government efforts to mitigate gender inequity (7 respondents); how certain ethnic groups (or castes) are treated in your country in general (15 respondents); and government efforts to mitigate ethnic inequity (16 respondents).

The stakeholders we interviewed spoke of salaries, preferred locations for working, and the nature of work in Indian hospitals as migration causes. Opportunities for specialist training and subsequent professional development were also noted as limited in India, especially for doctors. In the case of medicine, some stakeholders suggested that the rise of domestic contract-based (temporary) employment in the domestic public service encouraged doctors to look outside the country for better conditions of employment. The appointment of doctors on contract in the public service was originally thought to help keep costs down, but according to one respondent, this approach “did the greatest harm to the profession and the medical future of India” (P23, Hospital Medical Head).

Occupation-specific factors include lower salaries, whether in the public or private sectors, poor working /employment conditions, and the lack of government jobs (which were consistently seen as the most desirable type of employment). These factors were noted for their role in influencing nurses in particular to consider emigration; “the fact is that private sector nursing salaries are very low and service conditions are poor. Government jobs are not available. Therefore nurses migrate” (P10, Academic Director, p. 86). Although economic factors are typically cast in the literature as the most important reasons for emigration [5], in specific reference to nurses, one respondent noted that “the reasons for migration are not always purely financial… (nurses) look for safety, security and respect and dignity of their profession”(P49, Nursing Association Representative). There were several indications from respondents that lack of respect also had a role to play: “nurses are not treated very well in Indian hospitals, as compared to, say, USA or UK. They are not given the respect or the responsibilities which they could shoulder” (P38, Academic Director). In general, the low level of professional autonomy nurses experience in India adds to the desirability of migration opportunities to locations where professional development and skills promotion is available [30]; as one respondent noted, “The scheme of Nurse Practitioners, which exists in the USA, is not available in India” (P38, Academic Director).

Our scoping report and the stakeholders indicated that rising education costs and the international orientation of health worker training enhanced the proclivity for health worker migration. Although health professional training is primarily intended for Indian practice, whether in the public or private sector, the curriculum materials used in Indian training institutes our research team visited were often international English language. There is also a conscious acknowledgement of the need to prepare health workers for potential international experience: “We want people to migrate, but we don’t have a conscious policy to [promote that]. … [But] people will migrate and this is better for the state if they migrate with a better bargaining position” (R2, Senior Department of Health Official). Indeed, in some educational institutions, training was presented as specifically and exclusively geared toward overseas employment: “In X [training college], the whole effort was towards training nurses for foreign jobs” (P10, Academic Director). In these cases, entering the health care profession can itself be explicitly used as a means of accessing international migration opportunities.

Health professionals are often trained in an environment where employment options are regularly cast in both the national and international context; as one key stakeholder stated: “Outside India such as Western countries, even in Sri Lanka and Pakistan, pharmacists are highly recognized and given equal importance as doctors. We can say that many migrate because of issues of lack of job satisfaction and recognition by the society than for money” (R6, Health Professional Association Representative).

Some stakeholders indicated that many doctors seek overseas opportunities not because of poor working conditions and salary in India but in order to gain post-graduate training. They suggested that India’s highly bureaucratized public health sector creates limited opportunities for physicians to enroll in specialized training, which then delays the candidate’s progression through the ranks. “Migration is not an issue. It is not about migration, doctors go for a postgraduate position once passed out (graduated) in abroad. So more than migration we can say that they are going for higher studies that cause the shortage” (R4, Senior Government Department of Health Official). Some stakeholders indicated that those who gain specialist training are more likely to move into the private sector once trained. These observations reveal that doctors and nurses engage in international migration for different reasons.

In terms of the central governments’ handling of migration, the general perspective of most stakeholders can be summed up in the following comment: “(the) Government of India and Government of Kerala are not promoting migrating. We are not stopping also, it is a passive thing” (R9, Senior Government Official). The idea that there was no active governmental promotion of migration, nor was it prevented, was a widely held opinion among our respondents. As one stakeholder noted, this policy “neutrality” needs to be contextualized in terms of the economic benefit the Indian state will potentially receive from migration: “Government of India does not have any policy of trying to put limits on migration or trying to insist on minimum domestic service. The reason is that government feels the need for foreign exchange which these nurses will earn” (P10, Academic Director). The discourse of state benefits derived from health worker migration opposes the idea of the state attempting to prevent out-migration and suggests that a multiplicity of viewpoints exist in India regarding the outmigration of health workers. These positions compete for influence over local and central state policy debates.

The burden of personal debt that was taken on to gain training was also seen to feed the compulsion to go overseas, particularly in the case of nurses. To quote one respondent’s summary reasons for migration, “the major attraction for most of those who migrate …is to pay back loans taken for their education” (P5, Academic Director). This compulsion was driven by policy decisions that liberalized the health educational market, as was explained to us by an academic director in Delhi: “So long as the government keeps on giving No-Objection-Certificates to private businessmen to open Nursing Colleges, and they keep on charging very high fees from the Nursing students, how else can the students recover the cost of training except by going abroad?” (P10, Academic Director).

#### How has this changed over time?

Trends in migration of Indian health workers were thought to be changing. One stakeholder explained that there was a big exodus of nurses around 1993 and again between 2003 and 2009, but now, these flows have been reduced “to a trickle.” These particular flows were directed at the USA, Australia, Singapore, and the Middle East. Stakeholders in general agreed that the migration flow has reduced: “Migration of nurses to USA, UK, Australia, Canada has stopped since they have closed the doors. Even in the Middle East, they are starting their own nursing colleges, so maybe migration there will also come down” (P54, Senior Government Department of Health Official).

Reasons for this “change” or apparent decrease in health worker migration flows were offered including the lack of availability of US visas at the time of research, although one respondent did note “We are hearing that, migration of nurses to USA may be allowed once again, but there is nothing official yet” (P49, Nursing Association Representative). Other reasons for a decline in the scale of migration included reduced job opportunities in light of the Global Financial Crisis, improvement of nurses’ salaries in India following recent Pay Commission reviews, and improvement in working conditions.

### Consequences of health worker migration

#### Health workforce shortages: is migration the cause?

Health bureaucrats revealed general ambivalence about whether international migration is the major factor behind health workforce shortages; “if nurses go abroad, it is certainly a loss. But the numbers are not alarming” (P10, Academic Director). Rather, the benefits from remittances were seen to compensate for outmigration and stakeholders, as well as our scoping report indicated that the training system (especially in the private sector) was perceived as producing an excess of skilled health workers for this purpose. This reveals the presence of competing observations about the nature of the problem (if it is a “problem” at all) in the case of the international migration of health workers from India.

During the time of our research, specific information about health workforce shortages indicated that in Punjab, the required posts for doctors at the primary level of health care were filled, but 249 specialist doctor positions (surgeons, OB & GY (sic), physicians and pediatricians) at the secondary Community Health Centre level were not filled [39]. In Kerala, an excess supply of health workers was evident “(In) India, yes, [there are adequate employment opportunities but] in Kerala, not yet. Because Kerala cannot absorb the number of personnel it trains” (R2, Senior Government Department of Health Official). Nevertheless, a shortfall of specialists at CHCs was acknowledged in the Kerala case. These specific shortfalls in both Kerala and Punjab may be linked to the bureaucratic delays evident in the public sector appointment process of senior physicians, something key stakeholders also commented on.

The sectoral and regional health workforce density differences in evidence across India partly explain the ambivalent position of health bureaucrats when asked to comment on the influence of HWM on workforce shortages nationally. Stakeholders highlighted that vacancies often reflect not shortages but distribution issues between the public and private sectors and across rural and urban locations. Some stakeholders bemoaned particularly the lack of vacancies for nurses in the government sector, indicating that government sector jobs are readily filled. One stakeholder reported encouraging health professionals to migrate rather than to go to the private sector in India, arguing that “private sector service conditions are very poor… if they get some better opportunities [overseas], stay there for some time and [then] come back” (R5, Regulatory Body Representative).

Perceptions of shortages of nurses nonetheless were held and explained by “(the) mismatch between need and availability in the rural and urban India” (P28, Hospital Medical Head). This speaks to a characteristic of shortages common to many countries, the maldistribution of health workers in rural and remote areas, and failures to improve conditions which could help to retain skilled health workers in rural and remote areas. International migration is generally understood as only one part of the issue of overall maldistribution of health workers across India, especially in regard to the lack of physician specialists and poor rural health service provision.

The mismatch between effective system infrastructure and the health workforce mix is also part of the calculus of shortages: there may be enough doctors to serve the population, but “the doctors are terribly overworked and we would want to have more infrastructure and then more doctors” (P28, Hospital Medical Head). Shortages or excesses in one occupational category will certainly influence the workload of the other: “We have not created enough posts for nurses. Moreover when a doctor has to serve in an area where there is no nurse staff, [this results in a] heavy work load that actually aggravates the situation” (R4, Senior Government Department of Health Official).

Some stakeholders saw migration as less relevant to health workforce shortages in terms of quantity but more about quality, where migration results in the loss of valued skills and experience: “All the first rank holders and really good people are serving for other countries. That should not happen. There is no shortage but there is a quality problem” (R19, Regulatory Body Representative). Some stakeholders indicated they had no trouble hiring health workers, “I can recruit Junior Resident doctors, Senior Resident doctors and also posts of nursing orderlies and sweepers etc. as per government policy. We fill up these posts on a regular basis through open advertisements or on contract or on ad-hoc basis through walk-in interviews” (P23, Hospital Medical Head), and another said, “We get a lot of applications for these vacant posts; we do not experience any shortage of applicants” (P28, Hospital Medical Head).

For others, however, structural bureaucratic bottlenecks in the public sector represented another dimension of health workforce distribution that complicated effective workforce deployment. Some stakeholders noted that bureaucratic issues slowed down approval for some positions in the public (government) sector leading to both the perception and reality of shortages: “(there are) some specialties like Anaesthesia, Medicine and Radiology in which we are not able to get Junior Registrars (JR) and Senior Registrar (SR) doctors despite repeated efforts. We have suggested hiring them on part-time basis by Government as per market rates, - but approval is yet to come” (P23, Hospital Medical Head). Many doctors, despite good working conditions found in the public sector, were reported not to leave the country, but rather to move to the private sector. According to one stakeholder, “A significant number of senior registrar (SR) doctors leave to join (the) private sector but a larger number of junior registrar (JR) doctors migrate from private sector to public sector, and in the public sector, from smaller hospitals to bigger hospitals” (P23, Hospital Medical Head).

#### Loss of national investment

Some respondents thought that any migration of health workers from India produced negative effects for the country, but always positive benefits for destination countries, as the literature also suggests: “when these people migrate, the foreign country gets trained persons free of cost and our country loses skills which have been developed at public cost” (P28, Hospital Medical Head). There was also evident concern that the international orientation of some sectors of health worker training would become crisis ridden as overseas opportunities dry up, leading to structural flaws in India’s increasingly internationally orientated health education system:

“We are going to enter a huge crisis in the nursing education sector. The markets outside have dried up, even in the Gulf. … Many of these kids have studied by taking on loans, they are not going to get employment. The market will bid down their wage to very low. Then political pressure can then help me create some more posts for the nurses, but social ramifications will be very worse” (R2, Senior Government Department of Health Official).

Other perspectives of migration were those that produced negative impacts at the personal/familial level, rarely spoken about in the literature, including the fate of children and elderly parents who may remain in India and marital distress linked to migration and temporary separation: “(It’s) difficult to take care of parents. Marital ties may weaken, even sometimes leading to divorce.” (P49, Nursing Association Representatives).

#### Deskilling and loss of value through global circulation

One of the concerns consistently raised in the literature is that health professionals who migrate from India may practice below their skill level [40], and this is particularly germane for nurses, raising important gender considerations [41, 42]. Some stakeholders offered limited insight on deskilling. One mentioned that nurses already overseas preparing for or awaiting the results of their nursing exams would take lower category jobs (P2, Academic Director). Another stakeholder thought that there were structural barriers to Indians rising beyond middle-management level in the USA, and another that Indian General Nursing and Midwifery (GNM) Diploma holders were being treated only as Assistant Nurses in Saudi Arabia. Bilateral engagements between India and major receiving markets did appear to address deskilling processes, resulting in a policy response: “In Saudi Arabia, Indian GNMs were being treated as Assistant Nurses. The reason was that nurses from the Philippines argued that since they were at B.Sc. level, they should be one level above our GNMs. The Saudi government agreed. Ultimately, the Government of India took a decision to allow only B.Sc. nurses to migrate to Saudi Arabia.” (P10, Academic Director).

#### Return migration and re-integration

Lack of knowledge and data with regard to health worker migration prevented an accurate assessment at the time of research regarding where health workers were migrating, if they returned and what happened to them after they returned. Some stakeholders suggested there was very little evidence of return migration, and our survey findings supported this, since only 1.9% of the nurse respondents and less than 1% of the other health professions were return migrants. Others did comment on return migration, mostly in reference to nurses: “People go to USA and UK for professional advancement. There is no data that they came back*”* (R40, Hospital Medical Head). Migration was seen as part of a life cycle decision that included marriage and permanent migration: “Generally unmarried girls go abroad, they marry and settle abroad. So there is very little return migration ”(P46, Academic Director). Others reported that nurses who go to the United Kingdom of Great Britain and Northern Ireland, the United States of America, Canada, and Australia “do not usually come back. They take their families with them” (P10, Academic Director). Another thought that, “So many nurses want to come back from USA and UK,” (P54, Senior Government Department of Health Official) but did not indicate whether they ever did fulfill their wish to return. Unless they move onto other destinations, return migration was seen as inevitable for those in the Middle East; “nurses who migrate to Middle Eastern countries are not settling there. They come back*.*” (P49, Nursing Association Representative).

The practice and pattern of return migration did emerge as distinct for doctors versus nurses, with one respondent noting the favorable mobility that doctors can receive from the state: “Some state governments give doctors five years sabbatical to work abroad, so they are assured of jobs when they return” (P54, Senior Government Department of Health Official). However, all returning health worker migrants can face restrictions in re-entering their profession. Although anyone, including returning migrants, can apply for advertised vacancies (in the public sector), there is an age limit: 30 years for nurses and 50 years for doctors. For instance, the Government of India appoints General Duty Medical Officers (GDMOs) through the Union Public Service Commission but there is an upper age limit of 35 years (P28, Hospital Medical Head). Overseas experience and credentials may not be recognized by the professional regulators and employers, as noted in the case of nurses: “Returning nurses struggle a lot. They cannot get back their government jobs and have to accept lower paid private sector jobs” (P49, Nursing Association Representative).

Return to India is not always a gratifying experience, as one stakeholder expressed, “The situation of nurses returning, usually from the Middle East, is pitiable. Many of them have gained valuable experience but the government does not absorb them. Corporate hospitals may give them jobs because they are experienced nurses and the hospitals need some stable staff” (P10, Academic Director). Re-integration into the Indian health sector after a period abroad provides its own challenges: “it is not easy for those who return from abroad to get adjusted easily. I think the major challenges for re-integration are adjustment issues to a different way of life and medical practice, lack of networking, different profile and attitudes of patients etc.” (P23, Hospital Medical Head). Although it appears that there is relatively little return migration, there is some evidence in the literature, affirmed by our stakeholders, that suggests that some Indian health professionals are willing to return “if they can get appropriate position(s)”; otherwise, “a few corporate hospitals are accepting returnees. Some of these returnees continue to make brief visits abroad” (P2, Academic Director). Some stakeholders also noted that pharmacists who returned were able to operate independent chemist shops or pharmacies.

Overall, the examination of return migration was constrained by a limited sample of return migrant respondents. The limited survey responses indicate that of all health professionals, nurses are nearly twice as likely to be return migrants. In these cases, the main policy challenge appears to be re-integration. Some stakeholders indicate that most returnees go into the private sector where employment conditions are worse than the public sector and that generally, despite the suggestion that medical tourism might be hiring health professionals with western medicine exposure, our survey suggested that return migrant numbers were not significant. Clearly, the issue of return migration and professional re-integration into practice for health professionals are important. Such policy issues are vital to understand if we want to examine how “brain circulation” might actually be achieved in the case of the Indian health system [43].

### Policy responses to health worker migration

Our stakeholders traced many of the health workforce challenges they identified to a lack of policy more so than migration. For example, the perceived shortage of nurses in India is because “authorities (are) not serious about quick expansion of nursing education” (P46, Academic Director). As another commented, “State level policies are poor” insomuch as “there is no training reserve nor any organization to undertake the training load of so many workers” (P23, Hospital Medical Head). Many seemed resigned to the lack of such policy attention, noting as possible explanation that “the Government of India does not object to foreign agencies and governments recruiting Indians for foreign jobs” (P28, Hospital Medical Head). This also suggests that if the government was concerned about shortages and/or migration of health workers, policy to suppress recruitment and uptake of migration opportunities might be a sensible place to start.^1^

Although many of the stakeholders held positions that should suggest involvement in policy consultation, few indicated any formal policy engagement. They nonetheless offered several thoughts about policy responses to health worker migration at different levels of governance which we categorize here as the national/meso- and macro-/global levels.

### National meso-level policies

#### Addressing the Push Factors

Suggestions to address push factors included improved salaries in the public sector, although recent increases in public sector pay for health workers appeared to some stakeholders to be having the desired effect: “salary in government sector is much higher than earlier. So people are less eager to go abroad. The first priority is to get a job in the government sector. Only if that fails do they go for migration” (P46, Academic Director). However, reductions in the availability of jobs in the public sector, combined with a decline in migration, meant that “nurses work for lower pay and with poor working conditions in the private sector” (P46, Academic Director).

According to those who discussed nurses, with the exception of National (central) Pay Commission interventions that determine nurses’ pay structures and other conditions of services, there has been little in the way of administrative response to requests to improve working conditions and professional standards. To address nursing shortages, there are some indications that the government “tries to increase the number of nurses,” and if there is a shortage, “the appropriate policy is simply to train many more nurses” (P38, Academic Director). This leads to “Government say[ing] that more and more nursing schools and colleges should be opened.” (P42, Regulatory Body Representative). Doing so, however, returns us to a consideration of the privatization, affordability, and quality of nursing education.

#### Education, training, and return of service

Return of service has long been discussed in the international literature as one means to ensure that countries receive some benefit from the state investments they make in health worker education and training [44]. One respondent reported that “there is some attempt at Government level to insist that doctors must compulsorily serve in rural areas for a minimum period. But the proposal is yet to be finalised or implemented” (P28, Hospital Medical Head). This policy suggestion was linked directly to the expenditures of public money, “if a doctor has been trained on the basis of government subsidies, then the person concerned must either refund the money spent or else must serve for a minimum period. Subject to these conditions individual freedom of choice must continue” (P38, Academic Director).

**Fig. 6:**
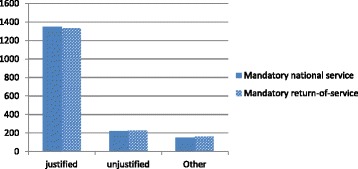
View of mandatory national and return of service (Source Health Professional Migration Survey, *n* = 1719)

**Fig. 7:**
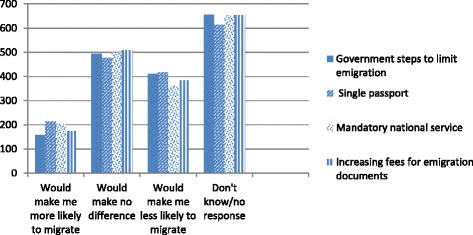
How would government policy effect your migration decisions? (Source Health Professional Migration Survey) *n* = 1719 each question

In the case of nursing, private sector employers had reportedly been confiscating nurses’ registration certificates and/or insisting on a bond period in order to retain their services (P49, Nursing Association). This practice was ruled illegal by the Supreme Court of India (ruling no. 527) in 2011. The Trained Nurses Association of India (TNAI) proposed a bill on nursing service conditions that would ban this practice and other types of service conditions. Policies that ensure mandatory national return of service were nonetheless seen by some stakeholders as necessary and justified: “We are spending so much on training. Those who are migrating must give an undertaking that they will come back and put at least some minimum years of service in India” *(*P17, Pharmacy Association Representative) (see Fig. 6). On the other hand, the majority of health workers surveyed indicated that such policy actions would make no difference to their migration decisions. However, the second highest level of health worker survey response does suggest policy actions to manage health worker migration might make some less likely to migrate, therefore achieving the presumed policy goal (see Fig. 7).

#### Mid-level cadres and new models of health care delivery

Alternative models of health care include the use of mid-level cadres of health workers who perform selected tasks under supervision. Such work would previously have been undertaken by highly qualified health professionals. Some stakeholders saw this occupational restructuring as a form of policy response in areas where shortages of doctors and nurses existed. In India, the use of mid-level cadres is fairly recent. One respondent explained, “The proposal to start a 3 year Bachelor of Rural Health Care is a good idea and it is now under examination by the Government of India…In the state of Chhattisgarh they have a cadre of Rural Medical Assistants. Similarly the state of Assam has Rural Health Practitioners” (P38, Academic Director) [45]. The same respondent explained that these qualifications are not transferable: “these are state-specific solutions and the graduates of these programmes are not entitled to practice outside the state concerned.” This also disqualifies them from migrating internationally as health workers because their qualifications and training are not transferable. This creates a fairly immobile occupational group whose qualifications may not be fully transferable across all Indian states, never mind internationally.

#### Encouraging return migration

How to encourage return and how to treat returning migrants poses domestic challenges: “if [returning migrants] are better than their domestic counterparts they should be paid more, but we should not in general pay more to a return migrant than to equally capable domestic counterparts” (P38, Academic Director). In the absence of policy that encourages the return of health worker migrants to India, “Government should create a working group to examine the factors contributing to [such] migration and to find ways of tackling these issues” (P49, Nursing Association Representative).

One policy suggestion related to return migration was the adoption of exchange programs, as one respondent explained: “Under these programmes a nurse goes abroad for a specified period, gets further training as well as employment for a fixed number of years and then returns to India” (P5, Academic Director). But, limitations of such international exchanges were pointed out: “The Western medical technology which some of the migrant nurses may have learnt to use, cannot always be adopted in India” (P5, Academic Director).

### Global and macro-level policies

At the macro-level, several respondents reported lack of knowledge or familiarity with regard to international codes such as the WHO Code, and its predecessors, the Commonwealth Code or the United Kingdom’s codes for the National Health Service [46–48]. The same level of knowledge was also reported for policies that encourage return migration or bilateral agreements between countries. One respondent referred to the “hidden” nature of the World Health Organization and lack of knowledge of the WHO Code, again suggesting that at some levels the international body has little visibility. Another person who did know about the codes was not able to pass on any knowledge with regard to their effects, while a third commented on their lack of impact. Despite some promise that these codes can help to “streamline the migration process” and that “India should take active interest” in them (P5, Academic Director), another complained that “the codes have no importance in practice. [The] government of India wants people to migrate because they get foreign exchange” (P32, Nursing School Representative).

### Health workforce data collection policy challenges

The knowledge of migration that our stakeholders shared was largely drawn from their own personal experience, and not from sources of official data. The lack of concrete data and statistics with regard to health shortages and migration consistently came up as explanation for lack of knowledge and an inability to answer some of the questions we posed. Routine information on India’s health workforce suffers from significant limitations [31]. It was a generally held observation that there is no government data on migration: “Nobody informs us about migration,” and no agency “is tracking the movement/migration of doctors and health workers across the states, regions, sectors or nations” (P17, Pharmacy Association Representative). The issues of data and data availability were not discussed extensively by those we interviewed, and there did not appear to be interest great enough to advocate for or encourage data collection at different points in a career trajectory: “No, why should we keep that [information]? Once they go out from college they are on their own” (R14, Academic Director). Some colleges did indicate some approaches to building limited databases on the location of their alumnae/i: “We know somewhat about who all have migrated. Other than that we don’t have data. It is quite difficult. We usually meet through Facebook” (R7, Academic Director). Another stakeholder noted that the “Indian Nursing Council (INC) is working to create a live register of members” (P49, Nursing Association Representative). In general, there was little to suggest much in the way of collection or coordination of data between state and national level bodies.

## Discussion

This paper offers an analysis of health care worker migration in terms of perceived causes and demographic factors associated with migration, an array of perceived consequences and appropriate policy responses in the case of India. Our research supports earlier findings regarding the migratory causes among Indian health workers, which includes push factors such as low remuneration, poor working conditions and work overload, concern with status of the profession, and lack of opportunities for professional advancement [2–6, 38]. Our research offered greater insight regarding the personal characteristics of those having applied for foreign work permits and licenses (more males rather than females, and in Punjab, those not currently married) and in terms of occupational sector (nurses exhibited higher rates of interest in migration than other professionals in our survey). Our research also revealed how the propensity to migrate varies according to sector-specific factors such as conditions of domestic employment and specialist training opportunities (physicians) and global cyclical recruitment trends for specific types of health workers (pharmacists). The detail our research offers suggests that determining who is most likely to engage in migration is a complex interaction of multiple factors shaped by an increasingly transnational context, this has not been fully explored in the literature in the case of India specifically, and for such a wide range of health worker occupations in general.

Our research highlighted the depth of contradictory thinking evident within key stakeholder communities with regard to the perceived influence of international migration of health workers on national health workforce issues. Stakeholder perceptions regarding whether migration caused shortages varied depending on which occupational group they were involved in and whether they were commenting on the wellbeing of migrants, access to care, or national economic development. The perceived drivers of migration and return migration for physicians, pharmacists, and nurses are identified as distinct, from seeking opportunities for specialist training, to increasing professional status and quality of life and responding to fluctuating international demand.

The scoping review concluded that India faces a myriad of challenges with regard to health system strengthening and improving basic access to primary health care in an equitable manner. The size of the population, the social and health disparities accompanied by demographic and epidemiological transitions that face this low- and middle-income developing country, and the pronounced disparities in access to health care, form the context in which a highly bureaucratized health system struggles to coordinate limited resources, including health human resources. Health worker migration is one of many factors that can contribute to health workforce shortages, but retaining health workers emerged as a lesser consideration for India’s attention [41].

In terms of the consequences of health worker migration, our research indicates that the government response to health worker migration at both the state and central government levels is marked by conflicting perceptions, something not previously examined in detail in the existing literature. While no official policies to promote international migration were identified in our interviews with key stakeholders, the benefits from migration that accrued to the state in terms of remittances, and the increasing role of the private sector in training health professionals for both domestic and international service, suggest a vested interest in this process of skills export does exist; this is an area for further examination as suggested in other studies [23]. Health worker shortages within India were often explained as consequences of factors other than migration, such as urban vs. rural and private vs. public workforce distribution issues that have been detailed before [11, 12, 25]. Our research, however, also revealed other health workforce policy failures and bottlenecks that have led to shortages and unfilled positions, especially in key specialist physician/surgeon and health management roles. Some direct consequences of international migration that were mentioned by key stakeholders included the social costs of migration (children and families left behind), the deskilling of professionals and the lost value that represents, and the challenge of re-integrating returning health workers into the domestic health sector.

Policy responses to health worker migration were discussed in terms of the national (meso) and global (macro) level. Improving the retention of Indian health professionals through government schemes to improve pay and conditions have limited influence in a context where the private sector is increasingly dominant, as has been detailed in the literature [17, 20, 23]. This lack of central government influence is most apparent in the case of nurses in the private sector, which appears to be less desirable than public sector employment. Likewise, national state action to increase training spaces and maintain health worker quality will likely be of limited consequence under conditions of increased private sector dominance without improved regulation of health training across all sectors [20, 23]. Physician-owned hospitals are among the private nursing schools exploiting nurses. Promoting deeper engagement with an interprofessional health team approach may help to form collaborative leadership between these occupational groups, potentially moving the system toward improved working conditions for nurses. The creation of new cadres of health care workers appeared as a solution to certain health worker shortages, but our research indicates the quality of this training and its ability to service national health needs demands further analysis, which would accompany recent inquiries on the matter [18, 19]. State policies to contain migration, such as bonding and return to service agreements, have been adopted in some cases, but their success is difficult to determine and in the case of bonding, has been ruled illegal by the Supreme Court of India. Based on our health worker survey results, the threat of various policies aimed at controlling migration (such as passport restrictions, increased emigration fees and return of service and national service) is relatively neutral.

The ability of the central government to exert control over health worker migration is not an area that has received significant research attention, and in light of the growing dominance of private sector training, any policy space the central government has in this area will likely diminish. Promoting return migration and encouraging international exchange and work experience did emerge as possible policy options, since they allow for international career development while maintaining connection to the domestic workforce. The use of international health worker mobility policies in order to retain workers in the long run has not emerged in the literature, and further research on this approach, and other forms of bilateral health worker mobility partnerships and trade in health services agreements, are areas of interest in terms of state-market policy responses to health workforce issues.

At the macro-level our key stakeholder interviews suggest there is relatively little knowledge of the Global Code on health worker recruitment and India’s position with regard to the Global Code [46–48]. International discourse on migration (WHO Code) is therefore not providing leadership. Aligned with relatively weak health data collection at the national and state levels [19], many of our stakeholder interviews revealed that the use of and contribution to any form of universal data collection was limited and appeared detached from health workforce planning and decision-making. This suggests that to assess the magnitude of the migration of health care professionals from India, the cooperation of destination country immigration and professional registration data collection systems is required. Without adequate data, of course, emerging patterns of health worker migration cannot be detected, making it more difficult to develop effective policies to manage migration processes. Perhaps, the move toward having National Health Workforce Accounts as part of the recently passed Global Health Strategy will assist in this regard.

This study was limited by a number of factors. Its geographical focus was on certain states (Kerala and the Punjab), which we caution may not represent experiences across India. There were two relatively independent survey research teams involved in data collection, and some differences in how the data collection was performed created some interpretive challenges for comparative analysis. The study’s cross-sectional design also precludes the possibility of longitudinal analysis. Our study however does make reasonable assumptions that survey and interview respondents knew of what they spoke and commented knowledgeably on the causes, consequences, and policy responses regarding health worker migration from India.

## Conclusions

Few studies have examined health worker flows and their consequences in detail and there has been an almost exclusive focus on the migration of medical and nursing practitioners and a lack of consideration of other highly skilled health professionals. Research to date has also given less attention to the range of policy responses that decision-makers at different levels have either taken or can select to stem the tide of emigrating health professionals or address domestic issues of health human resources distribution and shortages. This paper, based on an extensive and systematic scoping review of relevant literature, a health worker survey with 1719 Indian health professionals and 74 key stakeholder interviews (KSIs) provides in-depth analysis of these issues. Based on this research, we offer a number of conclusions which we cluster around three key points.

First, in addition to the standard economic reasons, the causes of health worker migration must also be understood in terms of the career opportunities and pathways open to the various health occupations; this is consistent with past research [30, 31, 38]. The negative consequences of poor planning in career development opportunities were especially evident in the lack of specialist training opportunities for physicians. Another factor that informs migration decisions is security of tenure, which appears more favorable for nurses and doctors in government rather than private sector employment, and appears to suppress the propensity to migration. Employment conditions in the private sector are poor for nurses and may encourage migration, in this case mostly for salary reasons. Doctors, on the other hand, appear more able to switch between the public and private sectors, thereby extracting beneficial conditions of employment by staying within India. Pharmacists and dentists appear to have benefitted from a growth in overseas opportunities in the last decade that have provided improved salary and training conditions overseas. The focus on career advancement and security of tenure, rather than just salary, suggests Indian health workforce planning and retention efforts must focus on system-wide dimensions of career development and security, in addition to compensation.

Second, we maintain that what appear to be the consequences of health worker migration are not easily separable from other causal and compounding factors. Shortages of health workers are evident in certain parts of the country and in certain specialty areas, but the degree and nature of such shortages are difficult to determine, and the strength of the relationship of such shortages to international migration is not clear. Our research suggests that shortages occur more because of shortcomings in domestic policy on training, recruitment and retention than as a direct consequence of the international migration of health workers. In these cases, international migration becomes an aggravating factor in the lack of staff in rural areas, but it is simplistic to see the one (international migration) directly causing the other (staff shortages). Rather, we echo the argument that international migration should be seen as compounding acute domestic problems in health workforce distribution [31, 38, 44].

Third, policy responses to health worker migration are embedded in wider processes aimed at health workforce management, but overall, there is not a clear policy agenda to manage or limit health worker migration at either state or central levels of government. Health bureaucrats hold conflicting opinions about the need or desirability of curtailing migration, and schemes that do place limits on emigration (such as Emigration Clearance Required for women migrants heading to certain countries) are pitched to address problems other than health worker shortages, such as dealing with fraudulent recruiters, securing personal protection or assurance of the employment rights and protections of emigrant workers. There are clearly tangible losses of investment made by the state and households, but within the wider context of diminishing opportunity structures and more privatized forms of human capital investment, it is hard to track where those losses become most acutely held. Based on the stakeholder interviews, it is fair to ask whether the Indian central and state governments might enable the process through their passive acceptance of health worker migration.

## Abbreviations

B.Sc.: Bachelor of Science
CHC: Community Health Centres
GDMOs: General Duty Medical Officers
GDP: Gross domestic product
GNMs: General Nursing and Midwifery Diploma (holders)
HIC(s): High-income country (countries)
HRH: Human Resources for Health
HWM: Health worker migration
JR: Junior Registrars
KSI(s): Key stakeholder interview(s)
LMICs: Low- and middle-income countries
MeSH: Medical subject headings
NHP: National Health Policy
NRHM: National Rural Health Mission
OB & GY: Obstetricians and gynecologists
RSBY: Rashtriya Swasthya Bima Yojana
SR: Senior Registrar
TNAI: Trained Nurses Association of India
WHO: World Health Organization

## Footnotes

1 Recently, the government of India has curtailed the migration of nurses to some parts of the Middle East, but this has been spurred to address complaints of abuses linked to the private recruitment industry, not out of a concern about shortages of nurses in India. http://www.thehindu.com/news/cities/Thiruvananthapuram/issue-emigration-clearance-to-nurses-cm-tells-centre/article7189808.ece.
